# Prevalence and Impact Factors of COVID-19 Vaccination Hesitancy Among Breast Cancer Survivors: A Multicenter Cross-Sectional Study in China

**DOI:** 10.3389/fmed.2021.741204

**Published:** 2021-11-03

**Authors:** Xin Peng, Ping Gao, Qiong Wang, Hong-ge Wu, Yun-li Yan, Ying Xia, Jian-ying Wang, Fang Lu, Hong Pan, Yi Yang, Fan Liang, Lei Zhao, Jing Cheng

**Affiliations:** ^1^Cancer Center, Union Hospital, Tongji Medical College, Huazhong University of Science and Technology, Wuhan, China; ^2^Department of Breast Center, Hebei Cancer Hospital, Wuhan, China; ^3^Department of Thyroid Breast Surgery, Tongji Hospital, Tongji Medical College, Huazhong University of Science and Technology, Wuhan, China; ^4^Department of Thyroid Breast Surgery, Renmin Hospital of Wuhan University, Wuhan, China; ^5^Department of Thyroid and Breast Gland Surgery, Zhongnan Hospital of Wuhan University, Wuhan, China; ^6^Department of Thyroid Breast Surgery, Wuhan Central Hospital, Wuhan, China; ^7^Department of Infectious Diseases, Union Hospital, Tongji Medical College, Huazhong University of Science and Technology, Wuhan, China

**Keywords:** COVID-19, vaccination, breast cancer, vaccine hesitancy, vaccination rate

## Abstract

Cancer patients are at a high risk of being infected with COVID-19 and have a poor prognosis after infection. Breast cancer is one of the most common cancers. Since vaccination is an effective measure to prevent the spread of COVID-19, we studied the vaccination rate among breast cancer survivors and analyzed their characteristics to provide evidence for boosting the vaccination rate. The researchers conducted a multicenter, cross-sectional study on 747 breast cancer survivors from six hospitals in Wuhan city between June 5, 2021, and June 12, 2021. The self-administrated questionnaires based on relevant studies were distributed. The researchers then compared differences in characteristics among vaccinated patients, hesitant patients, and non-vaccinated patients. Moreover, they performed univariable and multivariable logistic regression analyses to identify potential factors associated with vaccination hesitancy. The researchers assessed a total of 744 breast cancer survivors −94 cases in the vaccinated group, 103 in the planning group, 295 in the hesitancy group, and 252 in the refusal group. The vaccination rate was 12.63% (95% CI 10.25–15.02%) and 37.23% (95% CI 27.48–47.82%) patients reported adverse reactions. The vaccination hesitancy/refusal rate was 73.52% (95% CI 70.19–76.66%), which was independently associated with current endocrine or targeted therapy (odds ratio [OR] = 1.52, 95% CI 1.03–2.24), no notification from communities or units (OR = 2.46, 95% CI 1.69–3.59) and self-perceived feel (general vs. good, OR = 1.46, 95% CI 1.01–2.13; bad vs. good, OR = 4.75, 95% CI 1.85–12.16). In the hesitancy/refusal group, the primary reason was “I did not know who to ask whether I can get vaccinated” (46.07%), the person who would most influence decisions of patients was the doctor in charge of treatment (35.83%). Effective interaction between doctors and patients, simple and consistent practical guidelines on vaccination, and timely and positive information from authoritative media could combat misinformation and greatly reduce vaccine hesitancy among breast cancer survivors.

## Introduction

Since December 2019, Wuhan, China reported a cluster of novel COVID-19 cases that were caused by severe acute respiratory syndrome coronavirus 2 (SARS-CoV-2) (1, 2). COVID-19 rapidly spread worldwide with high contagion and has been officially declared a global pandemic (3). As of June 12, 2021, about 170 million cases of COVID-19 were confirmed worldwide, including about 3.8 million deaths (4). Cancer patients are a population of specific interest during the COVID-19 pandemic. Treatment-related side effects and other underlying diseases might present an immunosuppressive state and malnutrition in cancer patients. Two reviews suggested that cancer patients were highly vulnerable to SARS-CoV-2 and poor prognosis after infection, including high risk of mortality and intensive care unit admission (5, 6). Based on the global cancer statistics of 2020, female breast cancer has become the world's most prevalent cancer, and its incidence ranked higher than that of lung cancer (7). The statistics suggested that a large number of breast cancer patients had the risk of COVID-19 infection.

COVID-19 vaccines were touted as a promising preventive measure to mitigate the spread of COVID-19 (8). To accelerate its development, more than 200 vaccine candidates were studied for their efficacy against COVID-19, and the process of relevant clinical trials was accelerated. At present, a number of COVID-19 vaccines worldwide have received emergency use authorization. COVID-19 vaccination is facing insufficient confidence, changing acceptance, and preference heterogeneity from the public (9, 10). The delay in acceptance or refusal of vaccination despite the availability of vaccination services is known as vaccine hesitancy (11). Vaccine hesitancy caused by concerns about the safety of rapidly developed COVID-19 vaccines has been a great challenge in the fight against the COVID-19 pandemic (12). Targeting populations at risk of vaccine hesitancy with customized measures based on their characteristics is needed.

Cancer patients should be prioritized for COVID-19 vaccination (13) as they are a vulnerable population. A recent study showed that most patients with cancer should be recommended to receive vaccines when possible (14). However, many COVID-19 vaccine trials excluded cancer patients to limit the data on safety and tolerance. Meanwhile, ongoing cancer-related treatment would make their health condition unstable. Both situations made cancer patients hesitant about receiving a COVID-19 vaccine. One report suggested that as many as 30% of cancer patients were vaccine hesitant (15, 16). The number of breast cancer patients was huge. Meanwhile, following an intramuscular vaccine, axillary lymphadenopathy, which is easily confused with the axillary lymph node enlargement caused by cancer, was observed (17).

Studying vaccine hesitancy among breast cancer patients had great significance. Recently, Villarreal-Garza et al. studied vaccine hesitancy among 540 breast cancer patients residing in Mexico by social media channels of non-governmental organizations and observed a 34% vaccine hesitancy rate (18). This study discussed the willingness to vaccination but did not report the vaccination rate and related side effects. In this cross-sectional study, we recruited breast cancer survivors admitted to six local tertiary hospitals in Wuhan city, China. We estimated the vaccination rate and related side effects, along with vaccine considerations and informative routine on vaccine hesitancy. The findings of this study would help target possible hesitant breast cancer survivors and provide evidence for customizing strategies to improve the vaccination rate.

## Methods

### Study Design, Setting, and Participants

There are about 30 tertiary and graded A levels in Wuhan city, China. In this study, six major hospitals, which covered most cancer patients, were selected for this multicenter, cross-sectional survey. From June 5, 2021, to June 12, 2021, an anonymous web-based questionnaire was distributed through WeChat (a popular social media platform in China) to collect data; information confidentiality was guaranteed to each participant. The study had been approved by the Ethics Committee of Wuhan Union Hospital, Tongji Medical College, and Huazhong University of Science and Technology (20210580). We recruited patients who were diagnosed with breast cancer and were older than 18 years. Participants submitted an informed consent form before their enrollment. The exclusion criteria included (1) cognitive impairment, which might affect judgment and questionnaire filling; (2) taking <90 s to fill out the questionnaire; and (3) logical error in reported data.

### Data Collection

Healthcare professionals working in the field of breast cancer reviewed the questionnaire for content validity. Moreover, we conducted a pilot study for feasibility and recorded the average time required to fill out the questionnaire. The questionnaire consisted of five parts: (1) demographic characteristics; (2) status and willingness toward vaccination; (3) side effects among vaccinated participants; (4) reasons for non-vaccination and the person who would influence your decision and consideration in being vaccinated among non-vaccinated participants; and (5) channels and preference of vaccine promotion.

The demographic characteristics criteria included sex, age, marital status (unmarried/married/others), education level (middle school and below/high school/junior college/bachelor's and above), number of members in the family, annual family income (yuan), medical cost (self-paid/insurance/others), duration of cancer (years), and self-perceived feeling (good/general/bad). We also queried the participants about current breast cancer-related treatments, such as endocrine/targeted therapy, chemotherapy, and radiotherapy. COVID-19-related experience, including the infection history of participants and vaccination status of family, friends, and colleagues, was also enquired.

In section Methods, the participants were required to report vaccine status (Yes/No). If yes, they should report the number of vaccination (1/2) and vaccine-related adverse reactions. If their answer was no, participants were asked to report willingness to vaccination in the future (planning/hesitating/refusal). In sections Results and Discussion, we posed questions to participants who hesitated or refused vaccination in the future about reasons for non-vaccination and the person who would influence their decision and considerations in vaccination. Finally, all participants were asked about primary sources of information regarding COVID-19 (television media/mobile media/family and colleagues/medical institutions/communities/others) and their preference for vaccine promotion (70% efficacy rate for preventing infection/30% failure rate for preventing infection/7 out of 10 people can avoid infection).

### Statistical Analysis

We performed statistical description and group comparisons for basic characteristics among four groups: vaccinated group, planning group, hesitating group, and refusal group. Categorical variables were described using frequencies and percentages. The age variable was categorized into three groups (<40, 40–60, and >60). We expressed continuous variables as means with SDs when normality was met or medians with interquartile ranges (IQRs) when normality was not met and tested the difference between groups in categorical variables using a chi-square test or Fisher's exact test. For differences in continuous variables, we applied Wilcoxon rank-sum test.

Based on the definition of vaccine hesitancy, the hesitancy group and refusal group were combined as the hesitancy/refusal group (*Y* = 1), a combination of the vaccinated group and planning group was defined as the non-hesitancy group (*Y* = 0). Both univariable and multivariable binary logistic regression analyses were performed to explore potential and independent factors associated with vaccine hesitancy, ordinal predictors were treated as nominal variables. We calculated the odds ratio (ORs) and corresponding 95% CI and *p*-value. And we used the Hosmer-Lemeshow test to check the goodness of fit for the multivariable logistic model with entering procession. Furthermore, reasons for un-vaccination, the persons who would influence their decision, and their considerations in the vaccine were plotted with a histogram among the defined vaccine hesitancy group (hesitating group and refusal group). Following this, we performed data analysis and visualization using IBM SPSS Statistics (version 22, IBM Corporation, Armonk, NY, USA) and Microsoft PowerPoint 2016. A two-sided *p*-value < 0.05 was considered statistical significant.

## Results

A total of 747 participants signed the informed consent form and completed the questionnaire. Three participants were excluded for taking <90 s; no logical error was found. Total of 744 participants were included in the final analysis. Out of the total, 99.60% of participants were female while the number of male participants was only three (Table 1). Their age ranged from 20 to 83, and the median age was 48 years old. In total, 90.32% of them were married, 67.61% had a high school and higher degree, and 79.47% lived with more than three family members. In total, 73.79% of participants reported an annual income of more than 20,000 yuan, while 91.94% stated that their medical cost was supported by insurance. About 80% of patients had lived with breast cancer for <3 years; only 58 (7.80%) reported bad self-perceived feel. In total, 64.92% of patients had recently undergone breast cancer-related treatments, mainly endocrine and targeted therapy (40.05%). About half of the patients learned about the COVID-19 vaccination from communities or units (58.74%) and found that family members, friends, and colleges who met the vaccination condition were not vaccinated (48.52%).

**Table 1:** Basic characteristics of breast cancer survivors.

**Characteristics**	**All patients** **(***N*** = 744)**	**Vaccinated group** **(***N*** = 94)**	**Non-vaccinated group (*****N*** **= 650)**			***P***
			**Planning vaccination** **(***N*** = 103)**	**Hesitancy group** **(***N*** = 295)**	**Refusal group** **(***N*** =2 52)**	
Sex						1.000
Male	3 (0.40%)	0 (0.00%)	0 (0.00%)	2 (0.68%)	1 (0.40%)	
Female	741 (99.60%)	94 (100.00%)	103 (100.00%)	293 (99.32%)	251 (99.60%)	
Age, years	48.00 (40.00, 54.00)	48.00 (42.00, 57.00)	50.00 (42.00, 57.00)	46.00 (40.00, 54.00)	47.00 (40.00, 53.00)	0.080
<40	188 (25.27%)	18 (19.15%)	20 (19.42%)	79 (26.78%)	71 (28.17%)	0.459
40~60	477 (64.11%)	64 (68.09%)	70 (67.96%)	186 (63.05%)	157 (62.30%)	
>60	79 (10.62%)	12 (12.77%)	13 (12.62%)	30 (10.17%)	24 (9.52%)	
Marital status						0.912
Unmarried	19 (2.55%)	3 (3.19%)	2 (1.94%)	8 (2.71%)	6 (2.38%)	
Married	672 (90.32%)	86 (91.49%)	91 (88.35%)	268 (90.85%)	227 (90.08%)	
Others	53 (7.12%)	5 (5.32%)	10 (9.71%)	19 (6.44%)	19 (7.54%)	
Educational level						0.144
Middle school and below	241 (32.39%)	26 (27.66%)	38 (36.89%)	88 (29.83%)	89 (35.32%)	
High school	142 (19.09%)	16 (17.02%)	15 (14.56%)	54 (18.31%)	57 (22.62%)	
Junior college	200 (26.88%)	32 (34.04%)	29 (28.16%)	89 (30.17%)	50 (19.84%)	
Bachelor and above	161 (21.64%)	20 (21.28%)	21 (20.39%)	64 (21.69%)	56 (22.22%)	
Number of members in family						0.388
1~2	152 (20.43%)	29 (30.85%)	22 (21.36%)	57 (19.32%)	44 (17.46%)	
3	275 (36.96%)	30 (31.91%)	41 (39.81%)	104 (35.25%)	100 (39.68%)	
4	156 (20.97%)	17 (18.09%)	20 (19.42%)	67 (22.71%)	52 (20.63%)	
5~	161 (21.64%)	18 (19.15%)	20 (19.42%)	67 (22.71%)	56 (22.22%)	
Annual income, yuan						0.745
<20,000	195 (26.21%)	20 (21.28%)	25 (24.27%)	78 (26.44%)	72 (28.57%)	
20,000~100,000	371 (49.87%)	51 (54.26%)	51 (49.51%)	140 (47.46%)	129 (51.19%)	
110,000~200,000	111 (14.92%)	12 (12.77%)	18 (17.48%)	49 (16.61%)	32 (12.70%)	
>200,000	67 (9.01%)	11 (11.70%)	9 (8.74%)	28 (9.49%)	19 (7.54%)	
Medical cost						0.45
Self-paid	39 (5.24%)	2 (2.13%)	6 (5.83%)	17 (5.76%)	14 (5.56%)	
Insurance	684 (91.94%)	87 (92.55%)	94 (91.26%)	269 (91.19%)	234 (92.86%)	
Others	21 (2.82%)	5 (5.32%)	3 (2.91%)	9 (3.05%)	4 (1.59%)	
Duration of cancer, year						0.003^b, c^
<1	275 (36.96%)	26 (27.66%)	40 (38.83%)	104 (35.25%)	105 (41.67%)	
1~3	326 (43.82%)	35 (37.23%)	45 (43.69%)	139 (47.12%)	107 (42.46%)	
4~5	77 (10.35%)	15 (15.96%)	11 (10.68%)	33 (11.19%)	18 (7.14%)	
>5	66 (8.87%)	18 (19.15%)	7 (6.80%)	19 (6.44%)	22 (8.73%)	
Self-perceived feel						0.024^d^
Good	231 (31.05%)	34 (36.17%)	42 (40.78%)	80 (27.12%)	75 (29.76%)	
General	455 (61.16%)	57 (60.64%)	58 (56.31%)	189 (64.07%)	151 (59.92%)	
Bad	58 (7.80%)	3 (3.19%)	3 (2.91%)	26 (8.81%)	26 (10.32%)	
Recent breast cancer-related treatments	483 (64.92%)	53 (56.38%)	67 (65.05%)	187 (63.39%)	176 (69.84%)	0.112
Endocrine/targeted therapy	298 (40.05%)	25 (26.60%)	36 (34.95%)	130 (44.07%)	107 (42.46%)	0.013^b, c^
Chemotherapy	134 (18.01%)	21 (22.34%)	17 (16.50%)	40 (13.56%)	56 (22.22%)	0.040^f^
Radiotherapy	51 (6.85%)	1 (1.06%)	9 (8.74%)	20 (6.78%)	21 (8.33%)	0.095
**COVID-19 related characteristics**						
Had been infected by COVID-19	6 (0.81%)	0 (0.00%)	2 (1.94%)	2 (0.68%)	2 (0.79%)	0.496
Inform from communities or units	437 (58.74%)	78 (82.98%)	67 (65.05%)	170 (57.63%)	122 (48.41%)	<0.001^a, b, c, e^
Existence of no vaccination in family, friends and colleges who met vaccination condition	361 (48.52%)	57 (60.64%)	54 (52.43%)	150 (50.85%)	122 (48.41%)	0.242

*Post-hoc comparison with Bonferroni adjustment:*

a *Vaccinated group vs. planning group*;

b *Vaccinated group vs. hesitancy group*;

c *Vaccinated group vs. refusal group*;

d *Planning group vs. hesitancy group*;

e *Planning group vs. refusal group*;

f *Hesitancy group vs. refusal group*.

We divided the surveyed participants into four groups: 94 cases in the vaccinated group, 103 in the planning group, 295 in the hesitancy group, and 252 in the refusal group. The differences in the basic characteristics of the four groups are presented in Table 1. The coverage rate of COVID-19 vaccination was 12.63% (95% CI 10.25–15.02%). Of the 94 vaccinated participants, 35 reported adverse reactions after vaccination (rate was 37.23%, 95% CI 27.48–47.82%), such as 27 cases of local reaction (redness, pain at the site of injection), 2 cases of systematic reaction (fever, fatigue, and headache), and 13 cases of other reactions. Six participants had been infected by COVID-19 previously, but none of them received the vaccination.

The vaccine hesitancy/refusal rate among the sample group was 73.52% (547/744, 95% CI 70.19–76.66%). We used univariable and multivariable logistic regressions to assess the association between basic characteristics and vaccine hesitancy/refusal. In the univariable models, the prevalence of hesitancy/refusal rate was significantly associated with age, years with breast cancer, self-perceived feel, recent endocrine or targeted therapy, and notification from communities or units. In the multivariable model, the *p*-value for the Hosmer-Lemeshow test was 0.62, suggesting an acceptable fit. After adjustment, age and years with breast cancer turned to be non-significant. Compared with good self-perceived feel, general and poor self-perceived feel increased the prevalence of hesitancy/refusal rate, OR = 1.46 (95% CI, 1.01–2.13, *p* = 0.045) and OR = 4.75 (95% CI, 1.85–12.16, *p* = 0.001), respectively. Current endocrine or targeted therapy and no notification from communities or units were also significantly associated with increased risk of hesitancy/refusal, OR = 1.52 (95% CI, 1.03–2.24, *p* = 0.034) and OR = 2.46 (95% CI, 1.69–3.59), respectively (Table 2).

**Table 2:** Univariable and multivariable logistic regression of characteristics for association with vaccine hesitancy.

	**Univariable**		**Multivariable^a^**	
**Characteristics**	**OR** **(95% CI)**	***P***	**OR** **(95% CI)**	***P***
**Age years**				
40~60	**0.65 (0.43, 0.98)**	**0.038**	0.71 (0.44, 1.13)	0.152
>60	**0.55 (0.30, 0.99)**	**0.046**	0.58 (0.29, 1.17)	0.127
<40	1.00		1.00	
**Marital status**				
Unmarried	1.00 (0.36, 2.82)	0.998	0.76 (0.24, 2.35)	0.628
Others	0.91 (0.49, 1.69)	0.755	0.99 (0.49, 1.97)	0.972
Married	1.00		1.00	
**Educational level**				
High school	1.29 (0.79, 2.11)	0.302	1.39 (0.81, 2.38)	0.231
Junior college	0.82 (0.54, 1.25)	0.36	0.97 (0.59, 1.57)	0.889
Bachelor and above	1.06 (0.67, 1.67)	0.807	1.21 (0.66, 2.21)	0.539
Middle school and below	1.00		1.00	
**Number of family**				
3	1.45 (0.94, 2.23)	0.091	1.29 (0.78, 2.14)	0.319
4	1.62 (0.99, 2.68)	0.057	1.45 (0.81, 2.58)	0.206
5~	1.63 (1.00, 2.68)	0.052	1.54 (0.87, 2.73)	0.142
1~2	1.00		1.00	
**Annual income, yuan**				
20,000~100,000	0.79 (0.53, 1.18)	0.255	0.86 (0.54, 1.35)	0.511
110,000~200,000	0.81 (0.47, 1.38)	0.44	0.93 (0.48, 1.80)	0.827
>200,000	0.71 (0.38, 1.31)	0.269	0.87 (0.40, 1.87)	0.713
<20,000	1.00		1.00	
**Duration of cancer, year**				
1~3	0.97 (0.67, 1.41)	0.878	1.02 (0.66, 1.59)	0.928
4~5	0.62 (0.36, 1.07)	0.086	0.76 (0.42, 1.40)	0.381
>5	**0.52 (0.29, 0.91)**	**0.023**	0.68 (0.36, 1.28)	0.228
<1	1.00		1.00	
**Self-perceived feel^a^**				
General	**1.45 (1.03, 2.05)**	**0.036**	**1.46 (1.01, 2.13)**	**0.045**
Bad	**4.25 (1.75, 10.33)**	**0.001**	**4.75 (1.85, 12.16)**	**0.001**
Good	1.00		1.00	
Endocrine or targeted therapy	**1.70 (1.21, 2.41)**	**0.003**	**1.52 (1.03, 2.24)**	**0.034**
Chemotherapy	0.89 (0.59, 1.35)	0.586	0.85 (0.51, 1.42)	0.542
Radiotherapy	1.51 (0.74, 3.08)	0.253	1.28 (0.59, 2.80)	0.533
**Medical cost**				
Self-paid	1.39 (0.63, 3.09)	0.413	1.41 (0.60, 3.27)	0.430
Others	0.58 (0.24, 1.43)	0.241	0.61 (0.24, 1.58)	0.310
Insurance	1.00		1.00	
Had been infected by COVID-19	1.39 (0.25, 7.66)	0.704	1.38 (0.23, 8.48)	0.725
No notification from communities or units	**2.44 (1.70, 3.49)**	** <0.001**	**2.46 (1.69, 3.59)**	** <0.001**
Existence of no vaccination	1.30 (0.94, 1.81)	0.111	1.27 (0.89, 1.81)	0.182

*OR, odds ratio; CI, confidence interval*.

a *Intercept = −1.56 (p = 0.417); Cox & Snellen r square = 0.08; Nagelkerke r square = 0.129; C statistics = 0.694. Bold values indicates that are statistically significant*.

**Figure 1:**
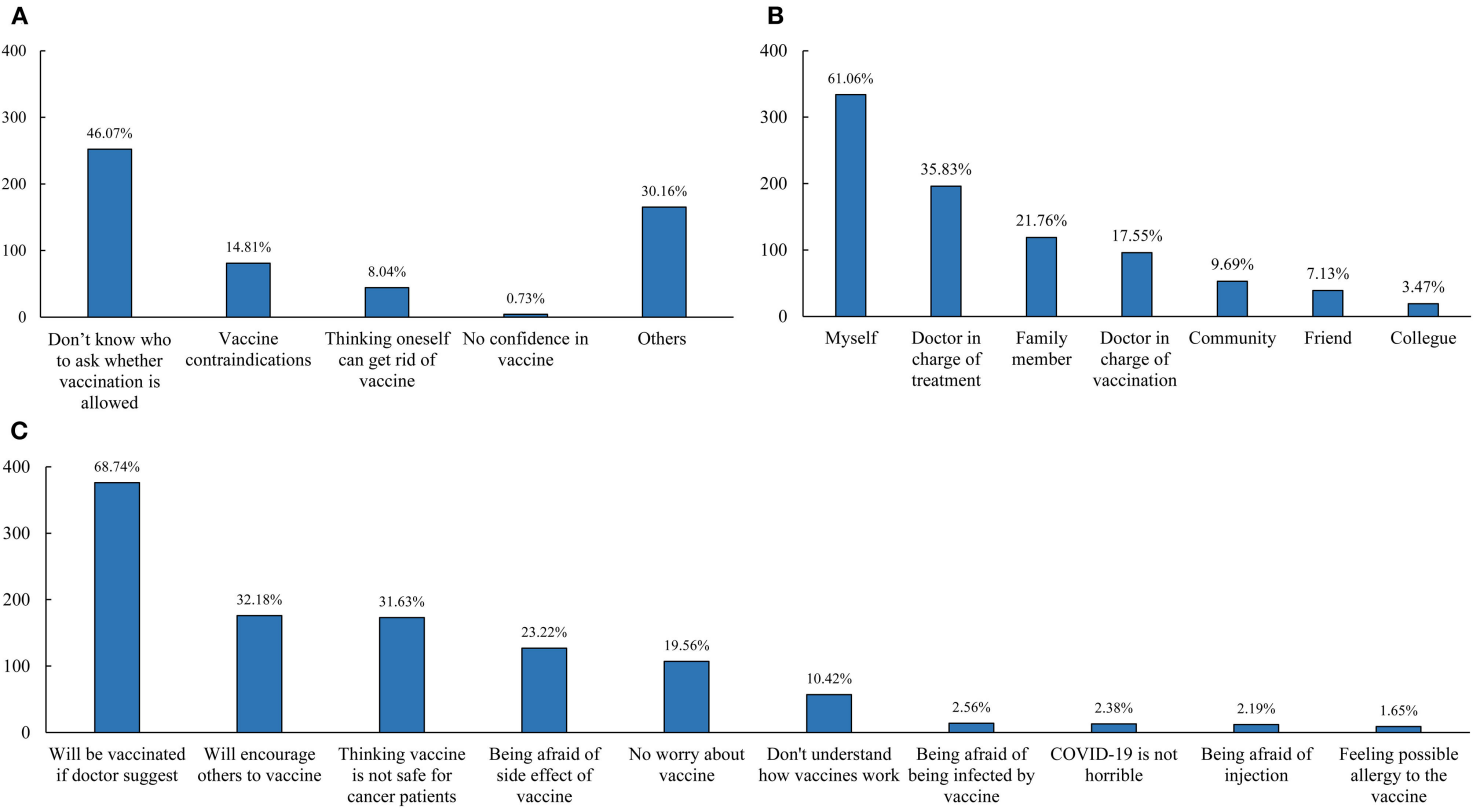
Reasons of vaccine hesitancy/refusal among breast cancer patients **(A)**, the person who would influence the decision of breast cancer patients **(B)**, and considerations in vaccine among participants with vaccine hesitancy **(C)**.

Furthermore, we explore the reasons influencing people and considerations regarding vaccines among 547 cases from the vaccine hesitancy/refusal group (Figure 1). The most common reason for vaccine hesitancy/refusal was lack of knowledge regarding the eligibility criteria (46.07%), followed by vaccine contraindications (14.81%), “think oneself can get rid of vaccine” (8.04%), and no confidence in the COVID-19 vaccine (0.73%), as can be seen in Figure 1A. Additionally, the opinion of doctors in charge of treatment (35.83%), family members (21.76%), and doctors in charge of vaccination (17.55%) influenced the decision of patients to vaccinate, as can be seen in Figure 1B. Regarding considerations of the COVID-19 vaccine (Figure 1C), 376 cases (68.74%) would get vaccinated if the doctor recommended and 176 (32.18%) would encourage others to get vaccinated, which suggested these cases were still waiting. In total, 31.63% of participants considered the vaccine unsafe for cancer patients, 23.22% were afraid of side effects, and 10.42% did not understand how the COVID-19 vaccine worked.

Finally, to promote COVID-19 vaccination, we collected channels of all participants for collecting vaccine-related information and preferred the wording for vaccine promotion. Mobile and television media were the primary sources for 67.88 and 51.75% of participants, respectively. The majority of participants (89.65%) preferred a “70% efficacy rate for preventing infection” for vaccine promotion.

## Discussion

The COVID-19 pandemic was still widespread in the country, while patients with cancer were at high risk of infection and poor prognosis. Vaccination is an economical and effective measure to prevent and control the pandemic. Based on recommendations from the National Health Services, cancer patients were recommended to get vaccinated after being fully informed and weighing benefits over risks (19–21). To our knowledge, this was the first multicenter, cross-sectional study to assess both vaccination rate and vaccine hesitancy/refusal rate among breast cancer survivors.

Various vaccines had been developed by different companies, such as Pfizer/BioNtech, Moderna, Johnson & Johnson's Janssen, and Sinovac and Sinopharm (two Chinese companies). Various protective efficacies and adverse effects in these vaccines with different platforms were reported (22, 23). The Chinese government had initiated the vaccination process on December 15, 2020. As of June 12, 2021, about 800 million doses of the COVID-19 vaccine have been administered, which suggested at least a 30% vaccination rate. However, the vaccination rate among breast cancer survivors was as low as 12.63%, which was similar to the vaccination rate worldwide (11.2%). In total, 37.23% adverse reactions were observed, and the common reaction was a local reaction at the injection site, which was slightly higher than the findings of two trials on the general population (24, 25). Meanwhile, no axillary adenopathy was observed even though a high rate of axillary adenopathy was reported after the administration of both the Moderna and Pfizer-BioNTech vaccines (21, 26). The low vaccination rate required effective and timely measures for improvement, and only high local reactions should be notified to reduce unavoidable anxiety and worries in vaccinators among breast cancer survivors.

Different levels of vaccine hesitancy had been found in the general public among 33 different countries (27). The nature of motives behind vaccine hesitancy could be complex, including such as policy and social factors, vaccine safety and effectiveness, and knowledge and experience of participants. In our study, researchers observed a high rate of COVID-19 vaccine hesitancy/refusal (73.52%) among breast cancer survivors, which was higher than previous studies on the subject in Mexico (34%) (18). The primary reason for vaccine hesitancy was that patients did not know whom to ask about the vaccination, which was different from concern about adverse effects in the study of Villarreal-Garza C et al. (18). Endocrine or targeted therapy, self-perceived feel, and notification from communities or units were identified as independent factors for vaccine hesitancy. Endocrine or targeted therapy is a long-term, complicated, and individualized treatment for breast cancer (28). The risk of adverse events related to the therapy was high, including the possibility of neutropenia and stomatitis (29). Both active treatment and adverse events would increase vaccine hesitancy in patients. Meanwhile, poor self-perception would decrease confidence of patients and willingness to receive the vaccination. It is notable that notifications about vaccines from communities or units were a powerful facilitator for vaccination. Communities and units were considered as communication centers to disseminate vaccine knowledge effectively.

The incidence of breast cancer varies greatly between male and female. Recently, significant differences between males and females in refusal of COVID-19 vaccination among general and cancer patients had been discussed (30–32). In our study, only three participants were male, and in the hesitant/refusal group, the influence of gender on hesitancy rate was not explored. To guarantee gender equality, the hesitancy rate and related factors in male breast cancer should be studied in the future. Moreover, dozens of COVID-19 vaccine candidates have been developed, and some vaccines with different protective efficacy were approved in an emergent way, potential and long-term side effects were not studied fully. The development of the COVID-19 virus might challenge the efficacy of existed vaccines. The unknown and variation could affect decisions of cancer patients and lead to varying degrees of vaccine hesitancy, which also required further studies.

In the hesitancy/refusal group, patients did not know whom to ask about the vaccination. Moreover, we found that opinions from doctors in charge of the treatment and vaccination could influence the decision of patients. A similar study (18) highlighted the same in its findings. Doctors play a central role in strengthening the confidence and trust of the public in vaccination. Maintaining an effective interaction and communication between healthcare professionals in charge of either treatment or vaccination of cancer patients could relieve their concern and address the high hesitancy rate (33). Individuals with different social, cultural, and individual backgrounds (34) showed no clear considerations. It is imperative to arrange for professional doctors to establish expert consensus or practical guidelines on vaccination. More importantly, an extended, simple, and clear patient-centered vaccination guideline should be constructed and distributed. This should include guidelines about conditions, precautions, contraindications, and possible vaccination reactions.

We found that mobile media were the primary source of COVID-19 vaccine-related information. According to the WHO, media and disinformation played a vital role in the resurgence of vaccine hesitancy, which is a major threat to global health (35). New media, such as mobile media and social media, offers mixed and multifaceted information. Moreover, it allows individuals to create and share unverified content quickly. People who hesitate or refuse vaccines were more likely to search for vaccine-related information on the Internet. Exaggerated reports of adverse reactions to the COVID-19 vaccine could result in a lack of confidence and panic among the public. Confusing and biased information further fuels vaccine hesitancy. Authoritative media and regulatory platforms should report COVID-19 vaccination information in a timely, positive, and accurate manner. This would guide patients toward learning and constructing verified knowledge about the vaccine and motivate suitable patients to be vaccinated.

## Limitations

Readers should consider the limitations of this study. Firstly, since it was a cross-sectional study, we restricted drawing any causal inferences. Secondly, due to resource limitations, breast cancer survivors from six tertiary grade A hospitals in Wuhan city were recruited. A large-scale survey is required to extend the generalization of our conclusion to other regions and countries. Finally, since the COVID-19 pandemic and vaccination are ongoing processes, attitudes of cancer patients to vaccines could change over time; a living survey system and updated guidelines for cancer patients should be developed and implemented to achieve this.

## Conclusion

Researchers observed suboptimal vaccination rates and high rates of vaccine hesitancy among breast cancer survivors in this study. It also concludes that endocrine or targeted therapy, poor self-perception, and no notification from communities or units can be used to identify the targeted population at high risk of vaccine hesitancy. Doctors in charge of treatment and vaccination can greatly influence these decisions of patients through effective interaction between doctors and patients, and simple and consistent practical guidelines on vaccination, timely and positive information from authoritative media could combat the misinformation and greatly reduce the vaccine hesitancy among breast cancer survivors.

## Data Availability Statement

The original contributions presented in the study are included in the article/supplementary material, further inquiries can be directed to the corresponding author/s.

## Ethics Statement

The studies involving human participants were reviewed and approved by Ethics Committee of Wuhan Union Hospital, Tongji Medical College, Huazhong University of Science and Technology. The patients/participants provided their written informed consent to participate in this study. Written informed consent was obtained from the individual(s) for the publication of any potentially identifiable images or data included in this article.

## Author Contributions

XP developed the idea, designed the study, and provided financial support for the study. XP and PG designed the questionnaires, drafted the manuscript, summarized the data, and contributed to data interpretation. QW, H-gW, Y-lY, YX, J-yW, FLu, HP, YY, and FLi were involved in the acquisition of the data. JC critically revised the manuscript for important intellectual content. The corresponding author had full access to all the data in the study and was responsible for submission for publication. All authors contributed to the article and approved the submitted version.

## Funding

The 2020 Huazhong University of Science and Technology Academic Projects (no. 2020kfyXGYJ001) supported this work.

## Conflict of Interest

The authors declare that the research was conducted in the absence of any commercial or financial relationships that could be construed as a potential conflict of interest.

## Publisher's Note

All claims expressed in this article are solely those of the authors and do not necessarily represent those of their affiliated organizations, or those of the publisher, the editors and the reviewers. Any product that may be evaluated in this article, or claim that may be made by its manufacturer, is not guaranteed or endorsed by the publisher.
